# Serotyping and pathotyping of *Glaesserella parasuis* isolated 2012–2019 in Germany comparing different PCR-based methods

**DOI:** 10.1186/s13567-020-00862-1

**Published:** 2020-11-17

**Authors:** Lukas Schuwerk, Doris Hoeltig, Karl-Heinz Waldmann, Katrin Strutzberg-Minder, Peter Valentin-Weigand, Judith Rohde

**Affiliations:** 1 Institute for Microbiology, Department of Infectious Diseases, University of Veterinary Medicine, Foundation, Hannover, Germany; 2 Clinic for Swine and Small Ruminants and Forensic Medicine and Ambulatory Service, University of Veterinary Medicine Hannover, Foundation, Hannover, Germany; 3 Innovative Veterinary Diagnostics Laboratory (IVD GmbH), Seelze, Germany

**Keywords:** *Glaesserella parasuis*, serotyping, pathotyping, leader sequence, virulence, serovar

## Abstract

*Glaesserella parasuis* is an important pathogen in swine production. It acts as a primary pathogen in systemic Glässer´s disease and as a secondary pathogen in Porcine Respiratory Disease Complex. In this study, a collection of 308 isolates from carrier animals and individuals with respiratory or Glässer´s disease isolated 2012–2019 in Germany was analysed. Isolates were characterized for serovar implementing two different PCR methods. Additionally, two different PCR methods for pathotyping isolates were applied to the collection and results compared. Serovar 6 (*p* < 0.0001) and 9 (p = 0.0007) were correlated with carrier isolates and serovar 4 was associated with isolates from animals with respiratory disease (*p* = 0.015). In systemic isolates, serovar 13 was most frequently detected (18.9%). Various other serovars were isolated from all sites and the ratio of serovar 5 to serovar 12 was approximately 1:2. These two serovars together represented 14.3% of the isolates; only serovar 4 was isolated more frequently (24.7%). The pathotyping method based on the leader sequence (LS = ESPR of *vta*) was easy to perform and corresponded well to the clinical background information. Of the carrier isolates 72% were identified as non-virulent while 91% of the systemic isolates were classified as virulent (*p* < 0.0001). Results of the pathotyping PCR based on 10 different marker genes overall were in good agreement with clinical metadata as well as with results of the LS-PCR. However, the pathotyping PCR was more complicated to perform and analyze. In conclusion, a combination of the serotyping multiplex-PCR and the LS-PCR could improve identification of clinically relevant *G. parasuis* isolates, especially from respiratory samples.

## Introduction

*Glaesserella* (*G.*) *parasuis* is a commensal bacterium of the upper respiratory tract of pigs [1, 2]. On the other hand, some strains of this bacterium can cause severe systemic disease, especially in weaner pigs, presenting as polyserositis, arthritis and meningitis (so-called Glässer´s disease, named after Karl Glässer, who described this condition in 1910 [3]). Additionally, *G. parasuis* is an opportunistic pathogen of the lower respiratory tract of pigs and involved in the porcine respiratory disease complex [4]. Initially, serovar was considered a major virulence trait [5]. However, conflicting reports on clinical symptoms caused by different strains of the same serovar, or in different pigs challenged by the same strain, shed doubt on a strict correlation between serovar and virulence [5–11]. Therefore, it became desirable to identify other virulence markers in addition to serovar, that can be determined in the clinical microbiology laboratory, in order to distinguish non-virulent commensals from potentially disease-causing virulent isolates.

In 2015 and 2017, PCR-based methods were published by different research groups both for convenient serotyping and for pathotyping of *G. parasuis*. Serotyping PCRs were both based on the sequences of the *cps*-locus but used different primer sets [12, 13]. The PCR designed by Howell et al. [12] reported an unspecific, additional band for serovar 2 and serovar 11 isolates, whereas no unspecific reactions were described for the PCR by Jia et al. [13]. Also, Jia et al. proposed to be able to distinguish between serovar 5 and serovar 12 based on a different gene of unknown function identified by whole-genome-sequencing.

Galofré-Milà et al. reported on an improved PCR for classifying virulent and non-virulent isolates based on the *vta*-locus, specifically the extended signal peptid region (ESPR), which they named leader sequence (*LS*)-*PCR* [14]. In contrast to this simple concept, Howell et al. published a *Pathotyping-PCR* based on 10 putative virulence-related sequences derived from genome-wide population studies [15]. Results of this PCR have to be analyzed by a specific software application to obtain a virulence classification for the isolate in question.

In the present study we applied both molecular serotyping methods and both pathotyping protocols to analyze a collection of 308 *G. parasuis* isolates isolated 2012–2019 in Germany from healthy and diseased pigs. Results were assessed in relation to the background information on the isolates as a silver standard for virulence since animal infection trails for such a large number of isolates (the gold standard for virulence testing) would not have been possible. Classification of isolates according to serovar reveals current frequencies of different serovars in Germany and both pathotyping methods result in useful information on the virulence of isolates based on in vitro markers.

## Materials and methods

### Bacterial strains

Initially the collection comprised 314 isolates. However, six isolates had to be excluded from the analysis after extended species analysis. A resulting total of 308 *G. parasuis* isolates with different clinical backgrounds, which had been collected between 2012 and 2019, were studied.

To obtain carrier isolates, 364 nasal swabs from 44 farms without any history of *G. parasuis*-related disease were cultured on chocolate agar with 0.07% NAD. From these swabs, 55 isolates were obtained and 29 of these 55 isolates were from different farms or of different serovar if from a single farm. These resulting 29 isolates were designated *carrier* isolates.

Another 22 isolates were cultured from nasal swabs of animals with lower tract respiratory disease and designated URT (*upper respiratory tract* isolates). Such nasal samples were submitted from veterinarians when bronchoalveolar lavage fluid or lung necropsy samples could not be obtained.

Isolates from bronchoalveolar lavage fluid or lung tissue of diseased animals were classified LRT (*lower respiratory tract* isolates, n = 204).

Finally, 53 isolates were obtained from brain, serous membranes or joint samples of animals with Glässer´s disease and assigned *systemic* isolates.

Information on the geographic origin of isolates is given in Additional file 1. Isolates were stored at − 80 °C before analysis. Control strains used in the four PCRs are listed in Table 1.

**Table 1:** **Control strains used in this study.**

Species	Strain	Serovar	*LS-PCR*	*Pathotyping-PCR*
*G. parasuis*	nr.4	1	V	V1, V2, V4, V6
*G. parasuis*	SW140	2	V	V1, V2, V3, V5, V9
*G. parasuis*	SW114	3	NV	V4, V7, V9
*G. parasuis*	SW124	4	V	V4
*G. parasuis*	Nagasaki	5	V	V1, V4, V5, V10
*G. parasuis*	131	6	NV	V3, V5, V7
*G. parasuis*	174	7	V	V1, V4, V10
*G. parasuis*	C5	8	NV	V3, V4, V7, V9
*G. parasuis*	D74	9	NV	V4, V5, V7
*G. parasuis*	H555	10	NV	V2, V7
*G. parasuis*	H465	11	V	V5, V6
*G. parasuis*	H425	12	V	V4, V9, V10
*G. parasuis*	84-17,975	13	V	V3, V4, V9, V10
*G. parasuis*	84-22,113	14	V	V4, V10
*G. parasuis*	84-15,995	15	V	V4, V5
*G. parasuis*	6694/5/18	6	NV	V2, V3, V4, V7, V9
*G. parasuis*	6868/5/18	2	V	V1, V2, V3, V6, V7, V8, V10
*G. parasuis*	6694/3/18	Non-typable	V	V1, V5, V6, V8, V10
*G. parasuis*	7013/4/18	7	V	V1, V5, V6, V9
*Actinobacillus (A.) minor*	8590/4/08		Negative	V3
*A. porcinus*	6416/3/04		Negative	Negative
*A. indolicus*	3753/11/08		Negative	V1, V3
*A. porcitonsillarum*	4931/04		Negative	Negative
*A. pleuropneumoniae*	ATCC 27,090		Negative	Negative

Markers underlined were used in different PCRs as positive controls.

### Identification of *Glasserella parasuis*

Species identification was carried out either by the PCR published by Oliveira et al. [16] or using the primers described by Howell et al. [12] in combination with the *LS-PCR* (s. below section “pathotyping”). Primers for these PCRs as well as all other primers used in this study are listed in Additional file 2.

### Serotyping

#### Serotyping according to Howell et al. [12]

The serotyping PCR described by Howell et al. [12] was split into two assays in order to have clear distinction between products of similar lengths. The first panel included the primers for serovar 2, 3, 6, 7, 9, 10, and 11, the second panel primers for serovar 1, 4, 5, 8, 12, 13, 14, and 15. PCR was carried out using Qiagen HotStart Taq DNA Polymerase (Qiagen, Hilden, Germany) and the standard reaction set up for a 25 µL reaction volume including a final concentration of 1.5 mM MgCl_2_ and 200 µM of each dNTP (Carl Roth, Karlsruhe, Germany). The reaction mix for the second panel included an additional 2 mM MgCl_2_ to improve signal strength. Primers (Biomers, Ulm, Germany) were used at a final concentration of 0.2 µM each. Cycling conditions were 15 min at 95 °C for activation of Taq, 30 s at 94 °C for denaturation, annealing was at 58 °C for 60 s and extension at 72 °C for 60 s. A final extension step at 72 °C for 10 min was programmed and a total of 30 cycles were run on a Bio-Rad T100 cycler (Bio-Rad, Munich, Germany).

#### Serotyping according to Jia et al. [13]

We did not succeed in combining the serotyping PCRs described by Jia et al. [13] as a multiplex PCR, neither by increasing the concentration of MgCl_2_ nor by adding 3% DMSO (NEB, Frankfurt, Germany) or 5 × Q-solution (Qiagen, Hilden, Germany). Individual serovar monoplex-PCRs were carried out with Qiagen HotStart Taq as described above. Final concentrations for primers were: for serovar 2—0.5 µM, for serovar 8, 9, 13, 15—1 µM, and for the serovars 1, 4, 6, 7, 10, 11, 14—2 µM. Only the PCR identifying serovar 5 and 12, respectively, was run as a duplex PCR with a final concentration of 0.2 µM for each of the serovar 12 primers and 1.2 µM for each of the serovar 5/12 primers. Annealing temperatures were chosen as listed by Jia et al. for the different primers and held for 90 s. 35 cycles were run. All other reaction components and cycling parameters were the same as described above.

### Pathotyping

#### LS-PCR according to Galofré-Milà et al. [14]

Establishing the *LS-PCR* followed the protocol by Galofré-Milà et al. [14]. In a multiplex PCR with the species-specific primers from Howell et al. [12, 15] each LS-primer (AV1-f, V1-r and NV1-r) was used at a final concentration of 1.5 µM and the species-specific primers at final concentration of 0.14 µM. Annealing was at 57 °C for 45 s and 30 cycles were run. All other reaction components and cycling parameters were the same as above.

#### Pathotyping-PCR according to Howell et al. [15]

Again, the *Pathotyping-PCR* as described by Howell et al. [15] was split into two multiplex PCRs. Conveniently, the 10 genes were numbered consecutively (V1-V10). Panel I encompassed V2, V3, V4, V7, V10 and panel II encompassed V1, V5, V6, V8, V9 (see also Table 2). Panel I showed better signals with 3% DMSO and additional MgCl_2_ (total final concentration 3.5 mM), panel 2 with additional MgCl_2_ in the master mix. Primer concentrations in the final reaction mix are listed in Table 2. The PCR was run at an annealing temperature of 54 °C for 30 s and with a total of 30 cycles. All other reaction components and cycling parameters were the same as stated above.

**Table 2:** Marker genes in the *Pathotyping-PCR.*

Name	Gene	Final primer concentration (µM each direction)	Panel	Marker association (according to Howell et al. [15])
*V1*	*HPS_21058*	0.40	II	Virulent
*V2*	*HPS_21059*	1.37	I	Virulent
*V3*	*HPS_21068*	1.14	I	Carriage
*V4*	*HPS_22970*	0.09	I	Virulent
*V5*	*HPS_23060*	0.19	II	Carriage
*V6*	*HPS_23300*	0.40	II	Carriage
*V7*	*HPS_23505*	0.09	I	Carriage
*V8*	*HPS_23879*	0.19	II	BAPS** 4 (virulent)
*V9*	HPS_23887*	0.52	II	Carriage
*V10*	*HPS_22976*	0.3	I	BAPS** 4 und 5 (virulent)

*Also named *HPS_23387* or *HPS_22887*.

**BAPS Bayesian Population Structure (Howell et al. [11]).

Results of the *Pathotyping-PCR* were supposed to be entered into an online tool [17], which, however, was out of service. Instead Kate Howell, the author, provided a software application in R-Studio (Rstudio Inc., Boston, USA), that had to be slightly modified, and finally expected results using a set of test data were obtained.

By this tool, isolates were classified into three categories: virulent, non-virulent and potentially virulent. According to Howell et al. the threshold for this buffer zone was chosen to minimize the false-positive and false-negative rates for their model [15].

### Sequencing

#### Cpn60 Sequencing

During the process of collecting isolates for this study, six isolates that were identified as *G. parasuis* using the Howell et al. primer pair [12, 15] could not be serotyped and did not show any product in the *LS-PCR*. Their *cpn60* universal target sequence was obtained and analyzed as described by Rohde et al. to inspect their species assignment [18].

#### Sequencing of the product of the LS-PCR

A single isolate showed two products in the LS- PCR corresponding to the virulent and non-virulent marker, respectively. Monoplex PCRs were repeated on a single colony of this isolate and PCR products were cleaned using a commercial kit (NucleoSpin, Machery-Nagel, Düren, Germany). Sanger sequencing was carried out by a commercial lab (Microsynth, Göttingen, Germany), results were trimmed and analyzed using SeqTrace 0.9.0 and aligned using clustalW [19].

#### Sequencing of the products of the Pathotyping-PCR according to Howell et al.

Since Howell et al. did not publish unequivocal reference strains for all 10 markers used in her PCR to classify isolates, we sequenced PCR products of the serovar reference strains and additional field isolates to define a set of control strains containing all 10 markers and to ensure specificity.

### Statistical analyses

Statistical analysis was done with SAS Enterprise Guide 7.1 (SAS Institute Inc., Cary, USA) using 2-tailed Fisher ´s exact test. The significance levels were as follows: 0.01 < *p* ≤ 0.05, significant, indicated by *; 0.001 < *p* ≤ 0.01, very significant, indicated by **; *p* ≤ 0.001, highly significant, indicated by ***. Confidence intervals (CI) of odds ratios refer to a confidence level of 95%.

## Results

### Species identification

Of the initial 314 bacterial isolates, 308 were identified as *G. parasuis* by the PCR described by Oliveira et al. [16] as well as with the primers published by Howell et al. [12]. However, six isolates were only assigned to this species using the primers designed by Howell et al. Additionally, these six isolates did not show a PCR product in the *LS-PCR* and could not be assigned to a serovar. Comparison of their *cpn60*-universal-target-sequences with published sequences of *G. parasuis* and other related *Pasteurellaceae* showed that they clustered more closely with isolates of *Actinobacillus* (*A.) minor* and *A. indolicus* (bootstrap values 0.9, see Additional file 3). Consequently, these six isolates were excluded from further analysis including pathotyping, which is known to produce also signals with *Actinobacillus* species [15].

### Serotyping

**Figure 1:**
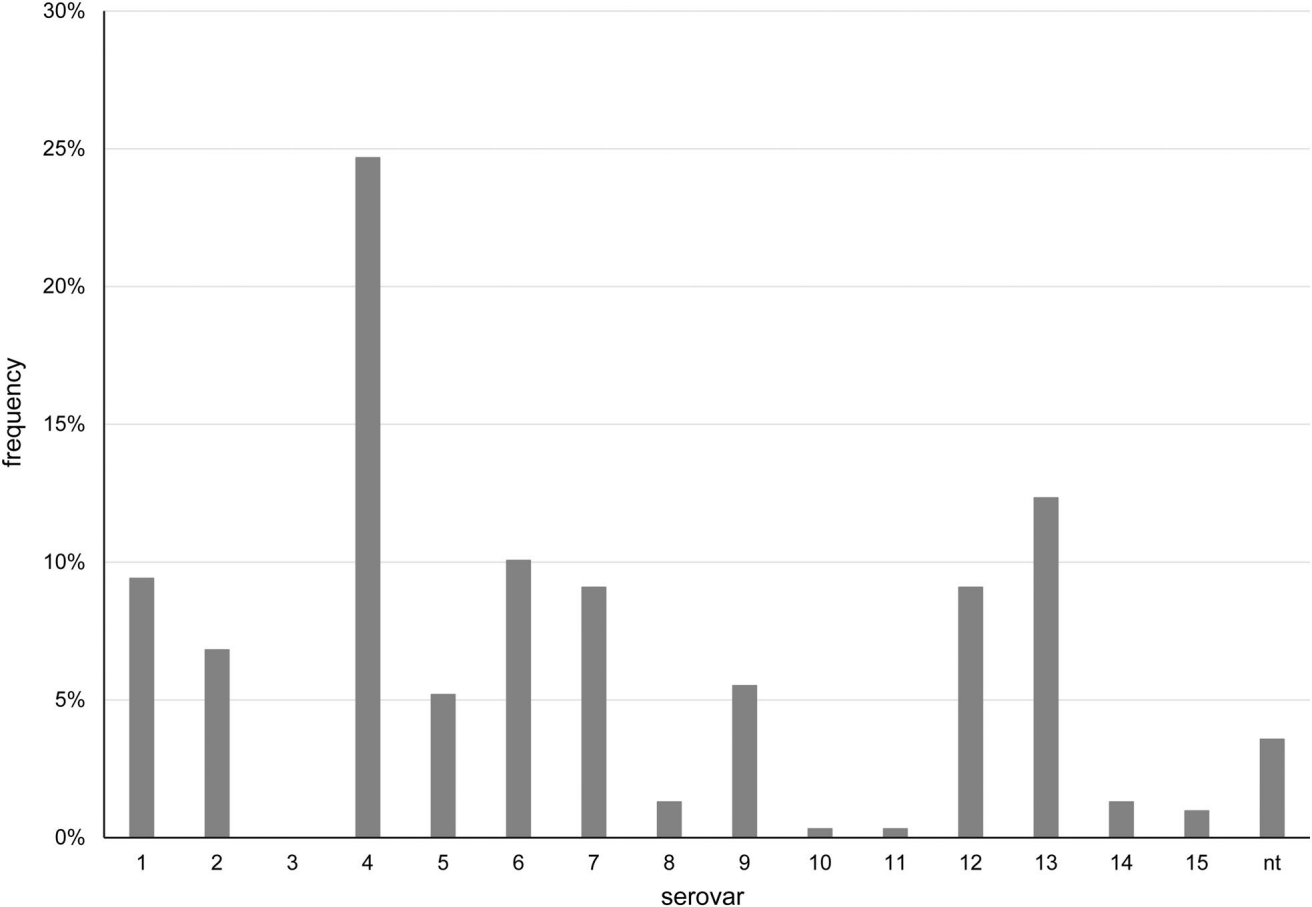
Serovars of G. parasuis isolated 2012–2019. n = 308; nt  = non-typable

Many different serovars were identified in our *G. parasuis* collection (Figure 1) with serovar 4 being the most frequent one (24.7% of all isolates). A number of different serovars were isolated at frequencies of 5.2–12.3% (serovar 1, 2, 5, 6, 7, 9, 12 and 13) while serovar 10, 11, 14 and 15 together encompassed only 2.9% of the isolates. A small proportion of 3.6% (11 isolates) could not be assigned to any serovar.

**Figure 2:**
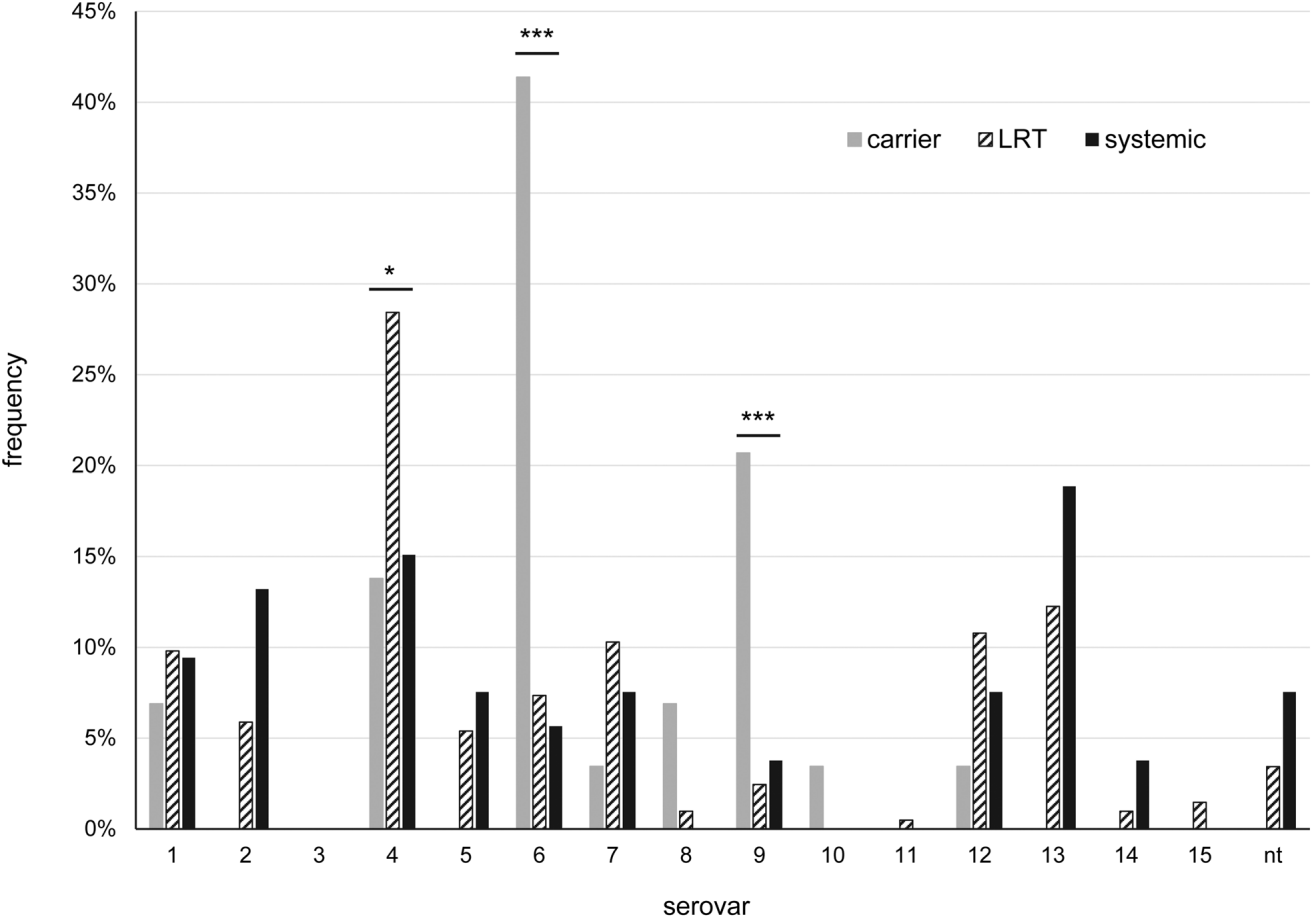
Serovars of G. parasuis in relation to clinical background. Presented are the frequency of individual serovars within the isolate groups carrier (n = 29), URT (n = 22), LRT (n = 204) and systemic (n = 53), respectively. nt = non-typable; *0.01 < *p* ≤ 0.05, significant; ***p ≤ 0.001, highly significant.

In relation to the origin of the isolates, most serovars could be detected in carrier, LRT as well as systemic isolates (Figure 2). However, serovar 6 (*p* < 0.0001; OR = 9.37 (CI: 3.89–22.61) with n = 286 excluding URT isolates) and 9 (*p* = 0.0007; OR = 9.32 (CI: 2.89–30.05) with n = 286 excluding URT isolates) were significantly associated with the carrier status whereas serovar 4 (*p* = 0.015; OR = 2.32 (CI: 1.17–4.59) with n = 286 excluding URT isolates) was significantly more often isolated from the lower respiratory tract of diseased animals. Among systemic isolates serovar 13 (18.9%), serovar 4 (15.1%), serovar 2 (13.2%) and serovar 7, 5 and 12 (7.5% each) were most frequently identified.

When isolates from nasal swabs from diseased animals (URT) were compared to isolates from bronchoalveolar lavage fluid or lung tissue (LRT), serovar distribution was very similar with one exception. Serovar 9 was significantly more often detected in URT isolates than in LRT isolates (*p* = 0.006, OR = 8.84 (CI: 2.18–35.88) with n = 226 for URT and LRT isolates).

Only Jia et al. described primers to distinguish serovar 5 and 12 [13]. With these primers 28 (60.9%) of the serovar 5/12 isolates identified using the PCR described by Howell et al. were assigned to serovar 12 and 16 isolates (34.8%) to serovar 5. Two isolates were not attributable to either serovar by the Jia et al. method. Additionally, 18 of the 28 isolates positive for serovar 12 were not amplified by the primers to simultaneously detect serovar 5 and 12 designed by Jia et al. but only by using the primers published by Howell et al.

Among the 251 isolates, which did not belong to serovar 5/12, only 20 showed discrepant or inconclusive results between the two PCR methods. Inconsistencies were confined to serovar 1, 2, and 11. Sixteen of these isolates showed the pattern described by Howell et al. for serovar 2 which includes an unspecific amplification of the serovar 1 marker (180 bp) in addition to the serovar 2 marker (295 bp) [12]. All of these isolates exclusively reacted with the serovar 2 primers designed by Jia et al. and consequently were classified as serovar 2 for the overall evaluation.

Two more isolates showed two PCR products in the Howell et al.-PCR, namely a 180 bp (marker serovar 1) and a 890 bp (marker serovar 11) product, and would be assigned to serovar 11 according to Howell et al. [12]. Both isolates reacted with the serovar 1- and with the serovar 11-primers designed by Jia et al., too. They were classified as non-typeable.

Finally, 2 isolates showed three bands in the multiplex-PCR (markers for serovar 1, 2 and 11) and were positive in the 2 monoplex-PCRs using Jia´s primers for serovar 2 and serovar 11. They were classified as non-typeable, too.

Though Howell et al. [12] saw amplification with the serovar 1 primers for serovar 2 and 11 isolates, there were five serovar 2 isolates and one serovar 11 isolate in our collection, that did not exhibit unspecific amplification of the serovar 1-marker.

### Pathotyping

Test key figures for both methods used for pathotyping isolates are summarized in Table 3.

**Table 3:** Key figures of the two PCRs used for virulence classification of carrier isolates (n = 29) and clinical isolates (LRT and systemic n = 257).

	*LS-PCR*	*Pathotyping-PCR*
Sensitivity	0.91	0.83
Specificity	0.72	0.67
Positive predictive value	0.97	0.96
Negative predictive value	0.49	0.29

#### *LS-PCR according to Gallofré-Milà *et al*. *[14]

One pulmonary isolate could not be assigned to a specific pathotype since it exhibited a virulent- and a non-virulent-specific PCR product at the same time. Sequencing of the two PCR products revealed a 99.5% match (one base substitution) with the reference sequence for a virulent isolate (EU678344.1 VtaA1 gene, strain Nagasaki) and 100% match with the reference sequence of non-virulent strain D74 (NZ_APBZ01000016.1 gene for YadA-like family protein, ESPR region), respectively.

**Figure 3:**
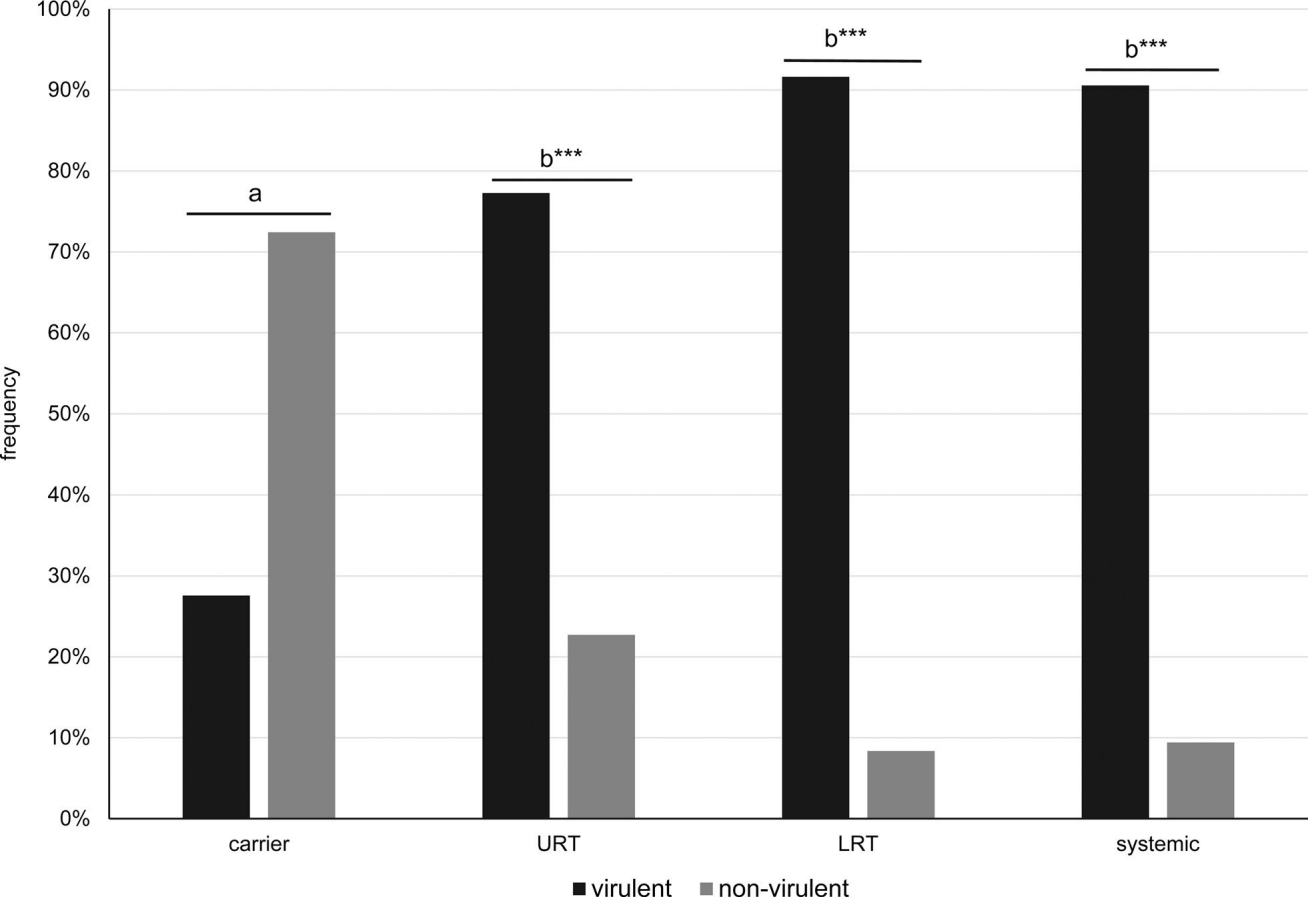
Classification of isolates into virulent and non-virulent by *LS-PCR* in relation to clinical background. Presented are the frequency of pathotypes within the isolate groups carrier (n = 29), URT (n = 22), LRT (n = 203) and systemic (n = 53), respectively.; different letters indicate statistical differences with ***p ≤ 0.001, highly significant.

Among all isolates, 84.4% were classified as virulent with the *LS-PCR* with significant differences for carrier and systemic isolates (Figure 3). Of the carrier isolates 72.4% were identified as non-virulent whereas 90.6% of the systemic isolates were assigned to the virulent class (*p* < 0.0001; OR = 25.2 (CI: 7.37–86.16) with n = 82 carrier and systemic isolates). This also applies to the comparison of carrier and LRT isolates with 91.6% of the latter being classified as virulent (*p* < 0.0001; OR = 28.72 (CI: 11.07–74.55) with n = 232) and to the comparison of carrier and URT isolates with 77.3% of the latter being classified as virulent (*p* = 0.0006; OR = 8.93 (CI: 2.46–32.34) with n = 51).

**Figure 4:**
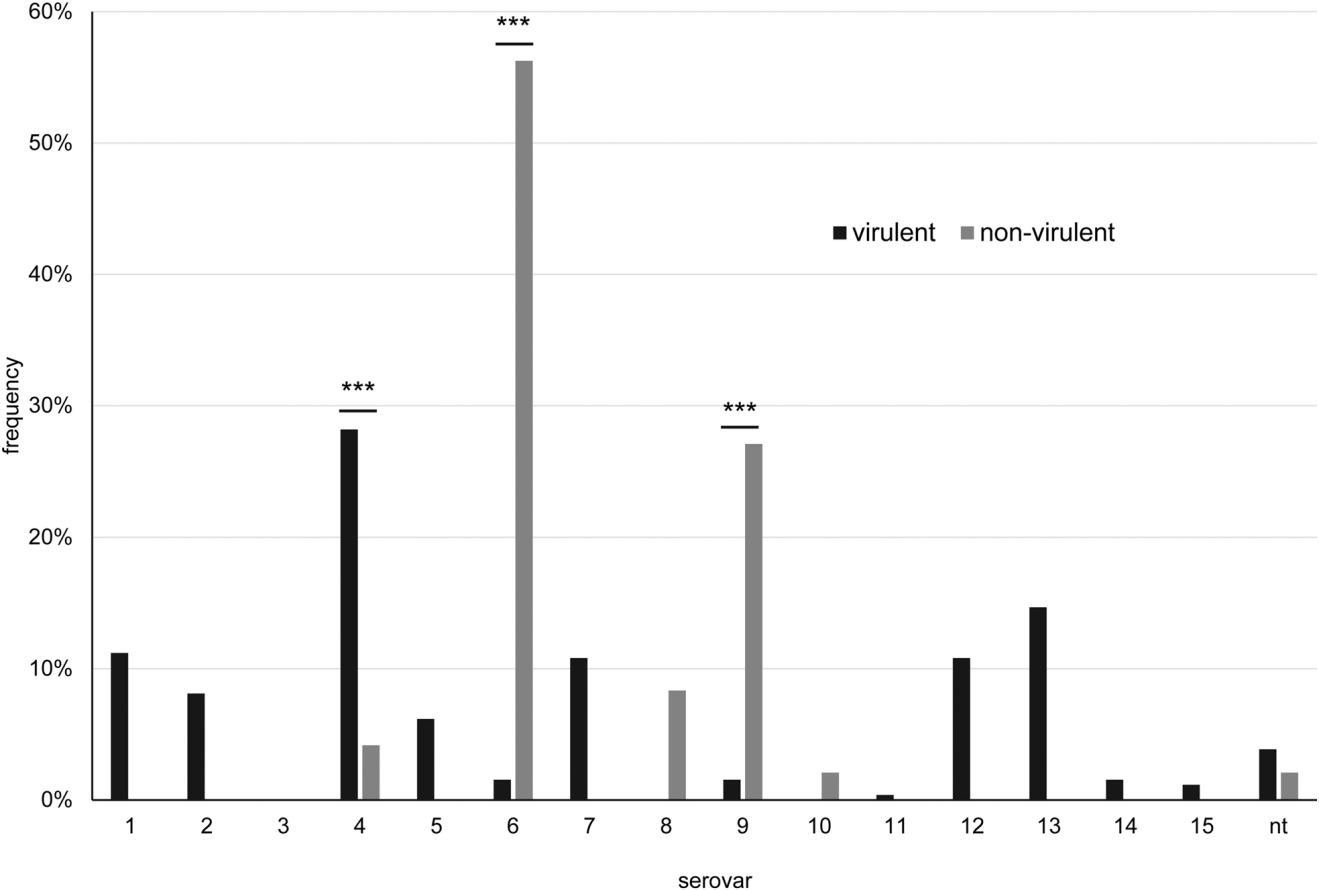
Frequency of individual serovars within categories virulent (n = 259) and non-virulent (n = 48) for LS-PCR. *nt*  non-typable; ***p ≤ 0.001, highly significant.

Isolates of specific serovars (namely 1, 2, 5, 7, 11, 12, 13, 14, and 15) were assigned solely to the virulent group, whereas serovars 8 and 10 were all classified as non-virulent (Figure 4). Serovar 4 was significantly associated with virulent isolates (*p* < 0.0001, OR = 9.03 (CI: 2.14–38.15) with n = 307), and only 2 isolates of this serovar were classified as non-virulent (one isolate from URT and one pulmonary isolate). In contrast, serovar 6 and 9 were significantly associated with non-virulence (*p* < 0.0001, OR = 81.96 (CI: 26.2–256.4) and *p* < 0.0001, OR = 23.68 (CI: 7.31–76.68), respectively, with n = 307) with all carrier isolates of these serovars being designated non-virulent. Notably, 10 out of 11 non-typable isolates were classified as virulent, whereas one was typed as non-virulent, but had been isolated from a pleura swab of a pig with pleuritis and pericarditis.

#### *Pathotyping-PCR according to Howell *et al*. *[15]

For the PCR products of the chosen control strains for V1, V2, V3, V5, V6, V7, V8, and V9 no mismatches were observed with at least one of the respective reference sequences [20], for V4 two, and for V10 nine mismatches were seen, respectively. Moreover, *Actinobacillus species* isolates used as controls showed bands for V1 and V3, respectively (Table 1).

**Figure 5:**
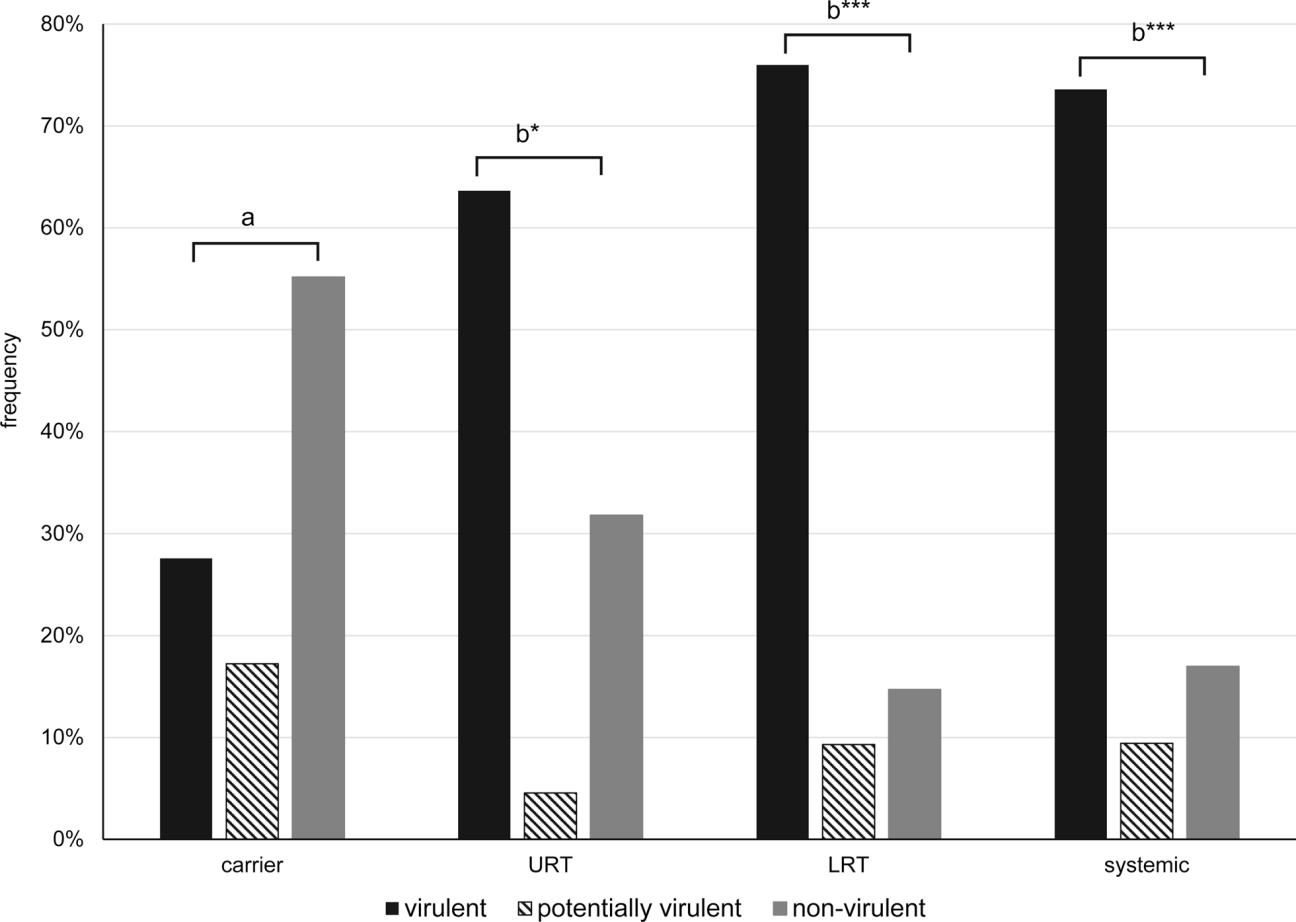
Classification of isolates into virulent, potentially virulent and non-virulent by Pathotyping-PCR in relation to clinical background. Presented are the frequency of pathotypes within the isolates groups carrier (n = 29), URT (n = 22), LRT (n = 204) and systemic (n = 53), respectively; different letters indicate statistical differences with *0.01* < p* ≤ 0.05, significant and ***p ≤ 0.001, highly significant.

The *Pathotyping-PCR* resulted in three classifications of isolates (Figure 5): virulent (70.1% of the isolates), potentially virulent (9.7%) and non-virulent (20.1%). Carrier isolates were more often classified as non-virulent (55.2%) while systemic isolates were more frequently identified as virulent (73.6%), which was statistically significant (*p* = 0.0001; OR = 8.67 (CI: 2.84–26.46) with n = 72). Similarly, LRT isolates also were statistically more often designated virulent (76%) compared to carrier isolates (*p* < 0.0001; OR = 10.33 (CI: 4.06–26.31) with n = 209).

Accordingly, when comparing the isolates from nasal swabs from animals without (carrier) and with disease (URT), respectively, isolates from diseased animals were significantly more often classified as virulent (*p* = 0.0377; OR = 4 (CI: 1.15–13.86) with n = 45). Fourteen (63.6%) URT isolates and 13 carrier isolates (44.8%) were characterized as virulent.

**Figure 6:**
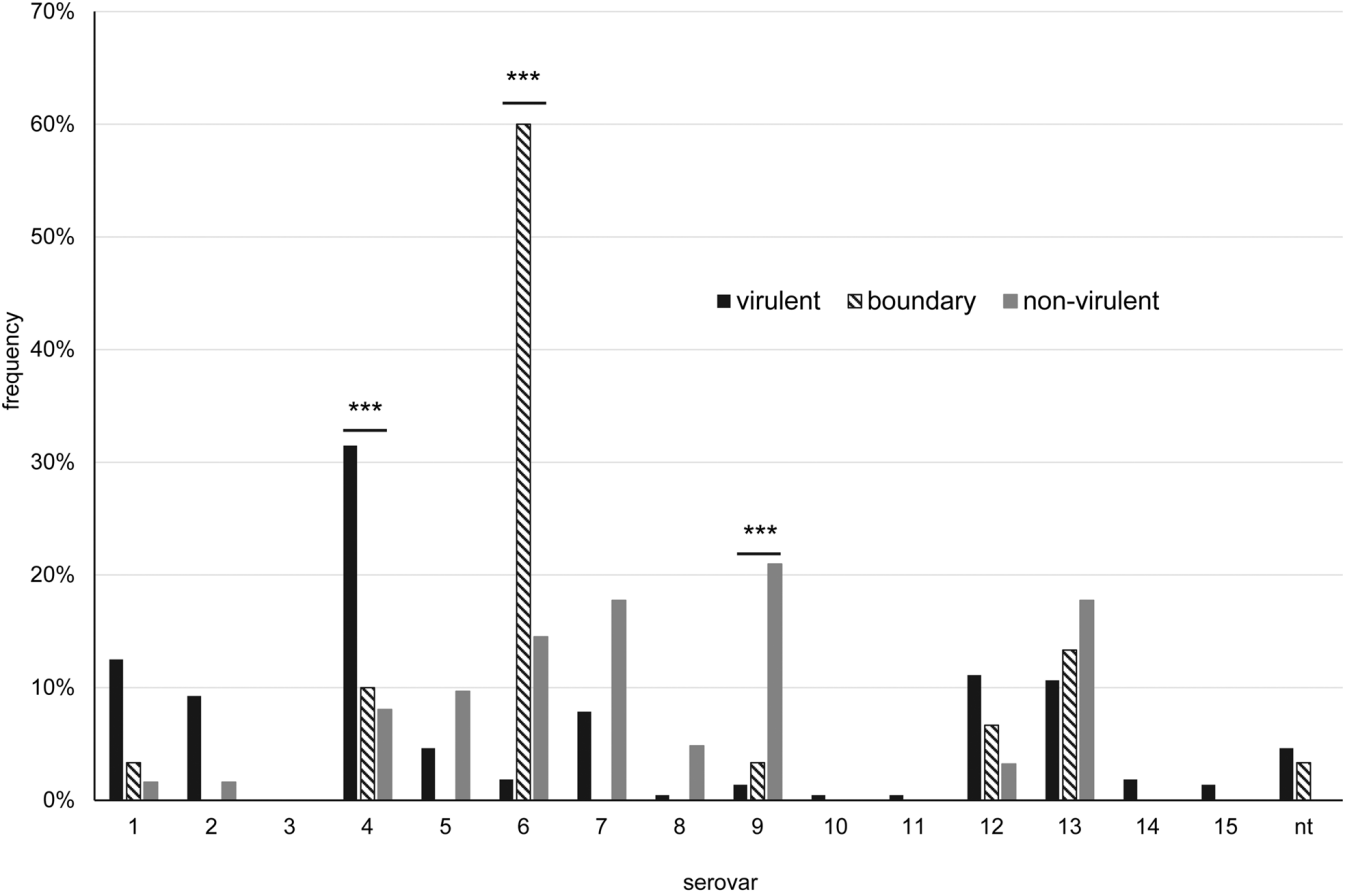
Frequency of individual serovars within categories virulent (n = 216), potentially virulent (n = 30) and non-virulent (n = 62) for the Pathotyping-PCR. nt=  non-typable; ***p ≤ 0.001, highly significant.

Results of the *Pathotyping-PCR* were less clearly co-related to serovars than results of the *LS-PCR*, since most serovars comprised isolates from different virulence categories (Figure 6). Again, serovar 4 included significantly more virulent isolates (*p* < 0.0001; OR = 4.82 (CI: 2.21–10.52) with n = 308) and serovar 9 significantly more non-virulent isolates (*p* < 0.0001; OR = 16,05 (CI: 5.02–51.3) with n = 308). In contrast, isolates with serovar 6 were mostly designated *potentially virulent* (*p* < 0.0001; OR = 30.58 (CI: 12.2–76.61) with n = 308).

#### Comparison of classification by LS- and Pathotyping-PCR

A total of 41 isolates were divergently classified by the two methods (14.7% with n = 277 see below). Most of these (n = 27; 65.9%) were from the LRT, which usually were classified virulent by *LS-PCR* and non-virulent by *Pathotyping-PCR* (n = 25). Moreover, seven systemic isolates, three URT and two carrier isolates followed this pattern. Only four isolates were identified as virulent by *Pathotyping-PCR* and non-virulent by *LS-PCR*, two from LTR and two carrier isolates.

In relation to serovar, eleven of the 41 divergently classified isolates belonged to serovar 7 and serovar 13, respectively, six isolates appertained to serovar 5, three isolates to serovar 4, two isolates each to serovar 9 and 12, and one to serovar 1 and 2, respectively. They were classified as virulent by *LS-PCR* and as non-virulent by *Pathotyping-PCR*. Only one isolate of serovar 6, 8, 9, and 10, respectively, was divergently identified as virulent by *Pathotyping-PCR* and non-virulent by *LS-PCR*.

These comparisons do not include the group of isolates assorted *potentially virulent* using the *Pathotyping-PCR* (n = 30; 9.7%) and the isolate that showed both markers in the LS-PCR since their status is inconclusive.

#### Detailed analysis of Pathotyping-PCR

While there is no straightforward inference of pathotype from the fragment patterns obtained by the *Pathotyping-PCR*, it is still interesting to look at the different virulence/ non-virulence markers in relation to the isolates in which they were detected. All 10 markers were seen in the three isolate groups (carrier, LRT and systemic). However, some of them were more frequent in specific isolates than others. V3 and V7 were significantly associated with carrier isolates (*p* < 0.0001; OR = 10.95 (CI: 4.73–25.35) and OR = 29.01 (CI: 10.93–76.96), respectively, with n = 286). In contrast, 50.9% of systemic isolates were positive for V10 which was a significant larger proportion than for isolates from other sites (*p* = 0.008; OR = 2.93 (CI: 1.59–5.4) with n = 286).

The relationship of virulence markers to certain serovars is more complex. V3 was associated with serovar 6 and 9 and both traits, V3 and these serovars, were especially seen in carrier isolates (*p* < 0.0001; OR = 16.55 (CI: 6.93–39.51) and *p* = 0.0005; OR = 6.27 (CI: 2.28–17.22) with n = 308, respectively). V10 was frequently seen in non-serotypable isolates which were exclusively isolated from clinical cases (LRT or systemic). In serovar 4 isolates, which were mainly isolated from LRT samples of diseased pigs, V1 was common (*p* = 0.0014; OR = 2.64 (CI: 1.49–4.68) with n = 308). However, in serovar 5 both types of markers, virulence-associated V4 and carrier-associated V9 according to Howell et al., were frequent (both p < 0,0001).

Interestingly, the *Pathotyping-PCR* did not produce any amplification product from 13 isolates. However, these were still classified as (highly) virulent by the software application with a predictive value of 0.99 (with 1 as highest possible value). They were obtained from carrier pigs (n = 2) as well as from diseased pigs (URT n = 1; LRT n = 9; systemic n = 1). Seven of these isolates belonged to serovar 4.

## Discussion

### Species identification

Correct species identification is important especially when isolates are serologically non-typeable or pathotyped by the method according to Howell et al. 2017. In both cases *Actinobacillus species* which are commensals of the respiratory tract might confound results [21, 22]. However, the primers for the species marker identified by Howell et al. [12] and originally used in their serotyping and pathotyping PCR erroneously identified six isolates in our collection as *G. parasuis* which could not be confirmed using primers designed by Oliveira et al. 2001 [16] and were indeed closely related to *Actinobacillus* species by *cpn60*UT sequencing.

### Serotyping

Since almost 30 years, this study is the first report on serotyping of a comprehensive collection of field isolates of *G. parasuis* from Germany, one of the world´s leading pig producing countries [5].

As in many other countries in Europe and all over the world [6, 7, 12, 13, 23–28], serovar 4 is strongly represented in the present study with a portion of approximately 25% of our isolates belonging to this serovar. Similarly to recent studies from Northern Italy, the UK and Southern China [12, 13, 23], serovar 4 is the most important serovar in our collection, too, whereas in other countries serovar 5 (or serotype 5/12 respectively) appears to be more frequent [7, 24, 27, 28]. However, eight other serovars (namely 13, 6, 1, 12, 7, 2, 9, and 5) were detected at frequencies of 12.3–5.2% each, and only serovar 3 was not isolated at all. In the above cited studies serovar 3 is also not or only rarely detected except for one study investigating a collection of nasal isolates from healthy animals [2]. Thus, a wide range of serovars appears to occur in pigs in Germany similarly to other countries [12, 13, 24, 29]. Serovar diversity is highest in carrier isolates and decreases in LRT and systemic isolates indicating differences in virulence. Only two of these many serovars, namely serovar 4 and 5, are covered by commercial vaccines available on the German market. However, it has been shown for serovar 1, 12, 13, and 14 that serovar 5 vaccines produce sufficient cross-protection [30].

In the present study, implementing a molecular typing method, only 11 isolates could not be assigned to any known serovar. This is in contrast to older studies using gel immunodiffusion or indirect hemagglutination [5, 7] and may well be attributed to the methodology as discussed for other pathogens [31]. However, there are also countries in Asia or South America where the proportion of non-typable isolates is large [26, 27, 32]. The studies from those countries were using the same molecular method which had initially been developed based on a collection of isolates mainly from the UK. Non-typable isolates from outside the UK may either encompass other serovars not yet described, or the primer binding sites in those isolates may differ substantially from the sequences derived from the European isolates.

Correspondence of both serotyping methods was good. However, the method described by Jia et al. could not be multiplexed which is a major drawback when it comes to routine testing in the clinical microbiology laboratory. Unlike described for the Howell-PCR, the primers designed by Jia et al. showed no unspecific amplification of the serovar 1 marker in serovar 2 isolates and were less prone to do so in serovar 11 isolates even so they bind in the *funB* gene, too. Due to the lack of sera, inconsistent PCR results could not be resolved by indirect hemagglutination as the gold standard method.

Different targets were used for the shared serovar 5/serovar 12 marker in the Howell- and Jia-PCR. While Howell uses the *wcwK* gene, Jia targets the *funK* gene, and this may explain why 18 out of 28 isolates eventually designated as serovar 12 based on the hypothetical gene discovered by Jia et al. were negative when using the Jia et al. serovar 5/12-primers but positive in the multiplex-PCR by Howell et al. However, the hypothetical gene, which is allegedly specific for serovar 12, makes for an interesting addition to the multiplex-PCR by Howell to resolve serovar 5 and serovar 12 isolates for epidemiological purposes. Interestingly, in our strain collection serovar 12 was twice as frequent than serovar 5. However, this could not be verified by indirect hemagglutination due to lack of sera.

### Pathotyping

While classification of isolates as virulent or non-virulent was not totally in accordance with their clinical vs. non-clinical background, this would not be expected either. Because of its high nutrient requirements, *G. parasuis* has a low tenacity in the environment and depends on the host for sustenance. Therefore, virulent isolates, too, need to colonize animals for their survival. Nevertheless, significantly more isolates from sick animals (URT, LRT, systemic) were classified as virulent compared to carrier animals (Figures 3 and 5). Specifically, there seemed to be an association of serovar 6 or 9, non-virulent, V3 and V7 positive isolates with the carrier status.

While initial reports additionally claimed serovar 7 to be non-virulent [5], isolates of this serovar were predominantly isolated from LRT and systemic locations in our study (25 of 28). Of these, 16 were classified as virulent by *LS-PCR* as well as *Pathotyping-PCR* and the remaining ones by *LS-PCR* only.

Virulent serovar 4 isolates seemed to have a tropism for the respiratory tract and were significantly more often found in samples from URT and LRT (Figure 2) as already described by Angen et al. [7]. Isolates of other serovars (serovar 13, 12, 7, 1, 2 and 5) found in this location were also detected from systemic sites and their presence in the lung may be transient as the lung also is the main portal of entry into the body for *G. parasuis* [33].

From nasal swabs from animals with lower respiratory tract infections (URT), many times non-virulent serovar 9 isolates (13.6%) which are otherwise predominant in carrier animals were obtained. Therefore, it is not advisable to collect nasal samples for diagnostics of lower respiratory tract disease, especially if isolates might be selected for autogenous vaccines.

Survival of *G. parasuis* in the lung is correlated with its resistance to phagocytosis by alveolar macrophages [34]. This seems to be linked to virulence associated trimeric autotransporters (*vta*) [35]. Differences in *vta*s, or more specifically their ESPR region, are the basis for recognizing isolates as virulent by the *LS-PCR*. Accordingly, 91% of the pulmonary isolates were categorized as virulent with this PCR (Figure 3) as opposed to only 76% of the isolates by *Pathotyping-PCR* (Figure 5). Some of the non-virulent ones may be contaminants from the upper respiratory tract (serovar 6 and 9). The other non-virulent isolates in the *LS-PCR*, two serovar 8 isolates and a serovar 4 isolate, may be from animals strongly predisposed to disease development by other factors.

From systemic sites, virulent serovar 13 isolates were most frequently isolated followed by virulent serovar 4, and virulent serovar 2 isolates. Also, serovar 1, 5, 7 and 12 were detected which all is in accordance with other studies from around the world serotyping systemic isolates [6, 7, 13, 23, 36, 37]. Only five out of 53 systemic isolates were classified as non-virulent by *LS-PCR*, four were serovar 6 or 9 and one non-typable. They may represent contaminants in cases where serosa was swabbed or may stem from animals heavily predisposed to disease by other factors. Interestingly, both serovar 6 and one of the two serovar 9 isolates were associated with the detection *Streptococcus suis* in these locations which probably was the primary cause of the disease symptoms in these cases. Nine additional isolates were classified as non-virulent or potentially virulent by *Pathotyping*. However, those belonged to serovars 1, 2, 4, 5, 7, and 13 otherwise related to virulence which sheds doubt on these classifications. In this context, it is interesting that V10 of the *Pathotyping-PCR*, which was significantly associated with systemic isolates, was also detected in six of these nine divergently classified isolates.

The population of systemic and LRT isolates could not be distinguished, neither by *Pathotyping* nor by *LS-PCR*. The *LS-PCR* classified 91.6% of the LRT and 90.6% systemic isolates as virulent and the average virulence score of the *Pathotyping-PCR* was identical for both groups (0.86). Only when looking at the individual markers of the *Pathotyping-PCR*, V10 seemed to be correlated with systemic isolates in our collection. This points to a major influence of host and environmental factors on the course of the disease and may also explain why animals infected by the same strain show different lesions [8]. In a genome-wide-association study (GWAS), which was the basis for designing the *Pathotyping PCR*, Howell et al., too, could not find markers specific for respiratory isolates and only two genes, *hsdR* and F537_gp36, were overrepresented in systemic isolates [11]. However, these were not included in the *Pathotyping-PCR* [15]. Interestingly, the same study found that the virulent type of the LS is one of only 10 virulence factors overrepresented in clinical isolates (pulmonary as well as systemic) whereas the non-virulent LS-type was one of only five factors more frequent in non-clinical isolates [11]. This supports the use of a LS-variant-PCR for pathotyping as designed by Galofré-Milà et al. [14].

Part of the deliberations behind the *Pathotyping PCR* was, that it should not rely on characterized virulence factors only, but also consider concepts of genome reduction and anti-virulence in bacterial evolution [38–41]. Accordingly, the panel encompassed five markers associated with the carrier status in the initial study population [15]. However, even though many genes were significantly negatively associated with virulence, a reduction of the genome could not be demonstrated in the GWAS and pseudogenes were avoided when designing the multiplex-PCR [11, 15]. In our strain collection two markers, V3 and V7, were strongly associated with the carrier status and the serovars typical for those locations, namely serovar 6 and 9.

Primer composition for the *Pathotyping-PCR* allowed for up to 10% mismatches to the genomes used for designing them by Howell et al. Predicted in silico patterns of markers and observed multiplex-PCR patterns only were identical in 57% of the isolates in the Howell strain collection with one to three bands difference in their remaining isolates. The model was corrected for these differences so that 78% of these isolates in their study population still resulted in the same virulence category as predicted from clinical metadata [15, 20]. When applying the method to our strain collection, we encountered multiple challenges. One was the lack of definite control strains and a panel of such strains was established including sequencing of resulting PCR fragments. Representative sequences showed good agreement with reference sequences provided by Howell with the exception of V10 that had nine mismatches [20]. However, twelve mismatches were also seen between the three reference sequences of this marker themselves. Other challenges encountered for our isolate collection may be due to variations in primer binding sites compared to Howell´s collection or unspecific amplification of genes and/or pseudogenes other than the intended ones. Also, chosen marker genes, which were derived from the accessory genome of mainly UK isolates, might differ or be missing in isolates from other geographic origins. Interestingly, 13 isolates negative for all markers in the *Pathotyping-PCR* were classified as virulent by the software application with a score close to the maximum score of 1. They belonged to a variety of serovars and to all sample types and were also categorized as virulent in the LS-PCR.

Thus, though the underlying concept of the *Pathotyping-PCR* is convincing, it still has many limitations. Additionally, test key figures (specificity, sensitivity, positive and negative predictive value) are better for the *LS-PCR* than for the *Pathotyping-PCR* (Table 3).

Notwithstanding, in our isolate collection high positive predictive values indicate that classification of isolates as virulent with either method is a meaningful information for the clinician. Overall, a combination of serotyping multiplex-PCR and *LS-PCR* could improve identification of clinically relevant *G. parasuis* isolates, especially from respiratory samples in which contamination with non-virulent carrier isolates is an issue. Moreover, when selecting isolates for autogenous farm vaccines it becomes important to potentially identify multiple serotypes on the farm and assess their relevance, provided sample collection and sample analysis have been conducted and evaluated appropriately.

## Supplementary information

**Additional file 1. Geographic origin of isolates.**

**Additional file 2.** **Primers used in this study.**

**Additional file 3.** **Maximum likelihood tree of** ***cpn60*UT sequences.**

**Additional file 4.** **Raw data for** ***G. parasuis*** **isolates in this study.**

## Data Availability

The raw data set is included as Additional file 4.

## Abbreviations

BAPS: Bayesian analysis of population structures
bp: base pairs
CI: confidence interval
dNTP: deoxyribonucleoside triphosphates
DMSO: dimethyl sulfoxide
ESPR: extended signal peptid region
G: Glaesserella
GWAS: genome wide association study
LRT: lower respiratory tract
LS: leader sequence
MgCl_2_: magnesium chloride
OR: odds ratio
PCR: polymerase chain reaction
URT: upper respiratory tract
VtaA: virulence associated trimeric autotransporter adhesin
YadA: *Yersinia* Adhesin A
