# Supplementary material for: Serotyping and pathotyping of Glaesserella parasuis isolated 2012–2019 in Germany comparing different PCR-based methods
Source: Vet Res. 2020 Nov 17;51:137. doi: 10.1186/s13567-020-00862-1 (PMC7673094; doi:10.1186/s13567-020-00862-1)
Supplement: Supplementary file 1 — Additional file 1. Geographic origin of isolates. [file 13567_2020_862_MOESM1_ESM.pdf]

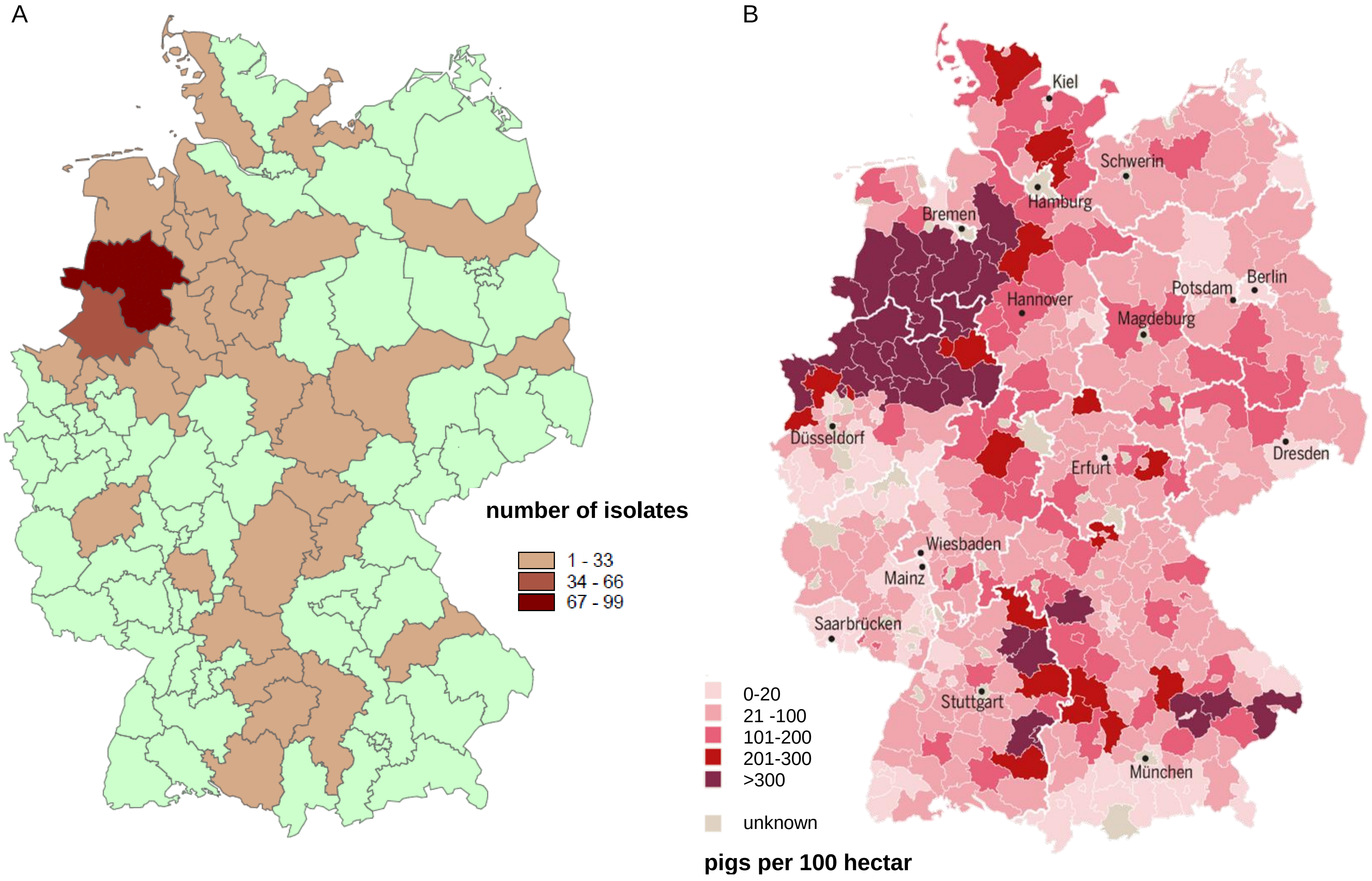

Geographic origin of *G. parasuis* isolates investigated in this study (A; the map was generated with our data using the software package "Das Postleitzahlen-Diagramm 4.0" by Klaus Wessiepe (www.Klaus-Wessiepe.de) licensed for „Institut für Mikrobiologie, Tierärztliche Hochschule Hannover", 2007) and pig population in Germany (B; map from Fleischatlas, 2016, by Heinrich-Böll-Stiftung, CC BY-SA 3.0 DE)
