# Supplementary figures and images for: Serotyping and pathotyping of Glaesserella parasuis isolated 2012–2019 in Germany comparing different PCR-based methods
Source: Vet Res. 2020 Nov 17;51:137. doi: 10.1186/s13567-020-00862-1 (PMC7673094; doi:10.1186/s13567-020-00862-1)

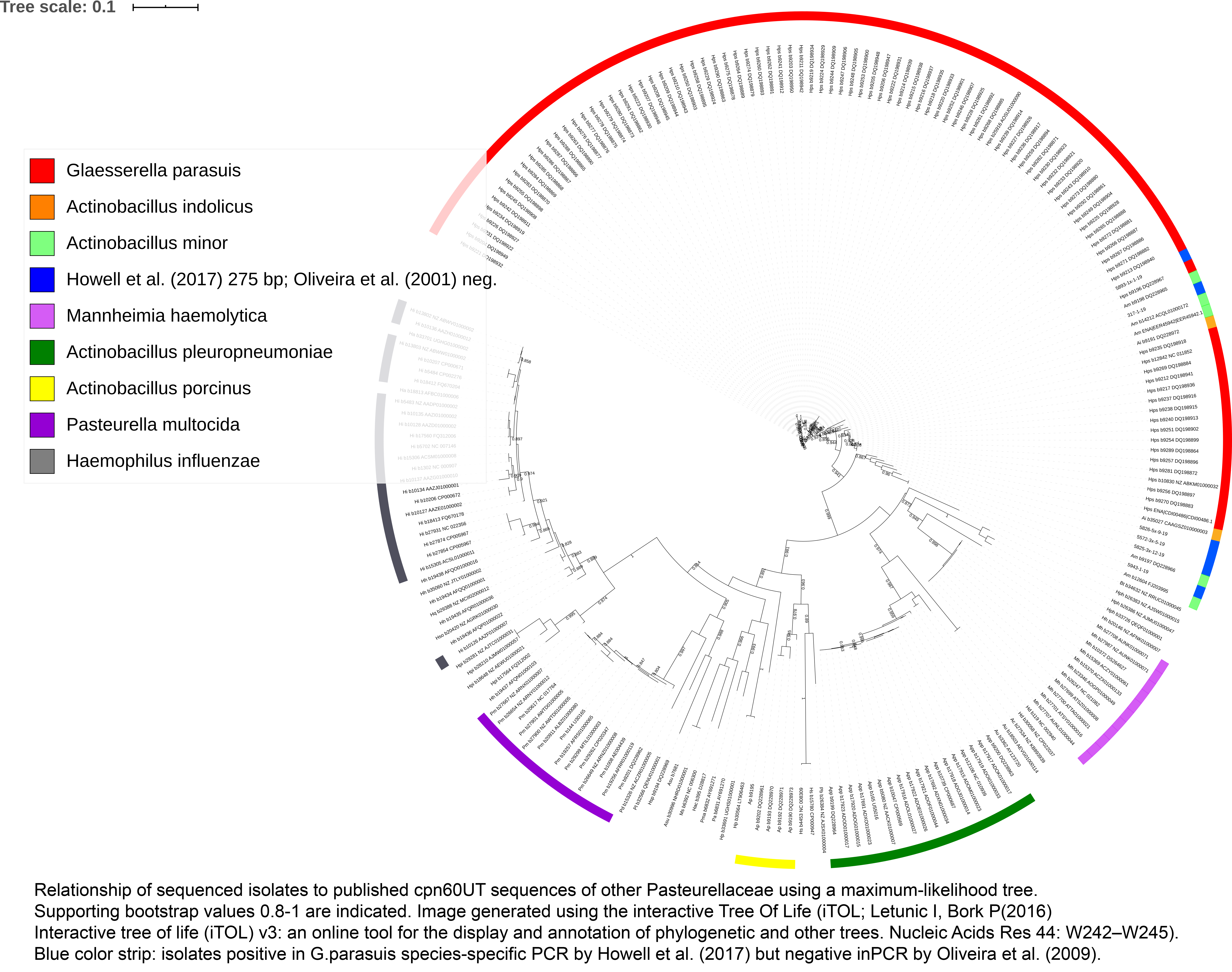

Supplement: Supplementary file 3 — Additional file 3. Maximum likelihood tree of cpn60UT sequences. [file 13567_2020_862_MOESM3_ESM.tif]
